# Comparisons of healthy human brain temperature predicted from biophysical modeling and measured with whole brain MR thermometry

**DOI:** 10.1038/s41598-022-22599-x

**Published:** 2022-11-11

**Authors:** Dongsuk Sung, Benjamin B. Risk, Peter A. Kottke, Jason W. Allen, Fadi Nahab, Andrei G. Fedorov, Candace C. Fleischer

**Affiliations:** 1 Department of Biomedical Engineering, Georgia Institute of Technology and Emory University, Atlanta, GA USA; 2 Department of Biostatistics and Bioinformatics, Emory University, Atlanta, GA USA; 3 Woodruff School of Mechanical Engineering, Georgia Institute of Technology, Atlanta, GA USA; 4 Department of Radiology and Imaging Sciences, Emory University School of Medicine, Atlanta, GA USA; 5 Department of Neurology, Emory University School of Medicine, Atlanta, GA USA; 6 Petit Institute for Bioengineering and Bioscience, Georgia Institute of Technology, Atlanta, GA USA; 7 Wesley Woods Health Center, Emory University School of Medicine, 1841 Clifton Road, Atlanta, GA 30329 USA

**Keywords:** Medical research, Neurology, Physics

## Abstract

Brain temperature is an understudied parameter relevant to brain injury and ischemia. To advance our understanding of thermal dynamics in the human brain, combined with the challenges of routine experimental measurements, a biophysical modeling framework was developed to facilitate individualized brain temperature predictions. Model-predicted brain temperatures using our fully conserved model were compared with whole brain chemical shift thermometry acquired in 30 healthy human subjects (15 male and 15 female, age range 18–36 years old). Magnetic resonance (MR) thermometry, as well as structural imaging, angiography, and venography, were acquired prospectively on a Siemens Prisma whole body 3 T MR scanner. Bland–Altman plots demonstrate agreement between model-predicted and MR-measured brain temperatures at the voxel-level. Regional variations were similar between predicted and measured temperatures (< 0.55 °C for all 10 cortical and 12 subcortical regions of interest), and subcortical white matter temperatures were higher than cortical regions. We anticipate the advancement of brain temperature as a marker of health and injury will be facilitated by a well-validated computational model which can enable predictions when experiments are not feasible.

## Introduction

Brain temperature is a key parameter correlated with cerebral activity. Several neurophysiological properties, such as intrinsic membrane properties, synaptic transmission, or blood–brain barrier (permeability, are sensitive to brain temperature changes^1–4^. Similarly, neural activation in mammals can result in local thermal fluctuations in the brain^5–7^. Higher brain temperatures are associated with worse outcomes for patients particularly after injury or illness, further emphasizing the importance of this relatively understudied parameter^8–12^. Given a lack of non-invasive tools to measure brain temperature in the clinical setting, body temperature is often used as a surrogate^13–16^. Magnetic resonance (MR) thermometry using the proton resonance frequency (PRF)^17–19^, among other temperature-sensitive parameters^20–24^, has been demonstrated extensively as a non-invasive approach in research applications. Despite the recent advances and clinical use of PRF thermometry, particularly phase-based mapping of relative temperature changes^25–27^, absolute temperature measurements using the chemical shift difference are still challenging due to long scan times^12,18,28^. The clinical utility of brain thermometry has been proposed for improved planning during targeted temperature management after cardiac arrest^15,29,30^ and patient stratification after ischemic stroke^11,31,32^, motivating alternatives to experimental measurements of absolute temperature.

Computational modeling validated through benchmark experiments is a viable alternative when direct experimental measurements are not feasible. Several bioheat transfer models have been developed^33^, most of which are based on the initial model derived by Pennes^34^. While the Pennes’ bioheat equation is the most widely used, it is largely simplified and based on uniform arterial perfusion, without consideration of advective heat flow within blood vessels or blood circulation through veins^35^. Chen and Holmes improved upon Pennes’ approach by adding directional blood flow to model advective heat transfer and convective heat exchange between tissues and blood vessels, and identified thermally significant blood vessels (i.e., vessels not in thermal equilibrium with surrounding tissue)^36^. Vasculature modeling was further improved by incorporating vessel curvature and branching in the discrete vascular algorithm (DIVA) model^37–39^. More recently, Shrivastiva and Roemer developed a model for perfused tissue based on principles of mass and energy conservation^40^, which was refined to reduce the high computational load^41^ and validated in a porcine model^42^. Our group recently demonstrated an improved model, building upon prior approaches^43–45^, which ensures local mass and energy conservation in formulation of the governing equations and is capable of personalized brain temperature predictions using individual input data from each subject.

Given the importance of brain temperature in assessment of both health and disease, and a lack of methods to routinely evaluate whole brain temperature in the clinical setting, a biophysical model capable of subject-specific predictions was developed in our previous pilot study. Comparison of model-predicted and MR-measured temperatures in a small healthy cohort was performed; however, complete statistical analysis and generalization were limited by the small sample size (N = 3)^46^. The goal of the present study is to evaluate the predictive power of our biophysical model^46^ applied in a larger cohort by performing regional analysis to compare thermal gradients and temperature patterns with those measured using whole brain chemical shift thermometry. Chemical shift thermometry was used for experimental temperature measurements as it is the only MR method capable of providing absolute and voxel-wise temperature values necessary for direct comparison with temperatures predicted using our model^18,20^. We anticipate the advancement of brain temperature as a marker of health and injury, particularly after brain injury or ischemia, will be facilitated by both experimental measurements and a well-validated computational model, which can enable predictions when experiments are not feasible.

## Methods

### Study participants and MR acquisition

This prospective study was approved by the local Institutional Review Board and all subjects provided written informed consent. All procedures were performed in accordance with the relevant guidelines and regulations. Inclusion criteria were medically healthy individuals of both sexes, any race, and any ethnicity, between 18 and 45 years old to avoid white matter changes due to age. Exclusion criteria were a history of neurodegenerative disease, epilepsy, ischemia, central nervous system surgery, moderate-to-severe traumatic brain injury, or contradictions to MR imaging. MR data was collected from 30 healthy volunteers, 15 males and 15 females (mean ± standard deviation [SD] age: 26 ± 4 years old; range 18–36 years old). Five participants self-reported their race as African-American or Black, 11 as Asian, and 14 as White or Caucasian. Two participants who identified as White also self-reported their ethnicity as Hispanic; all other participants self-reported their ethnicity as non-Hispanic. MR data was acquired on a 3 T MR scanner (PrismaFit, Siemens, Erlangen, Germany) using a 32-channel phased array head coil. A T1-weighted magnetization-prepared rapid gradient-echo (MPRAGE) sequence was used to acquire high resolution structural images (repetition time [TR]/inversion time [TI]/echo time [TE] = 2300/900/3.39 ms, flip angle (FA) = 9°, field of view [FOV] = 256 × 256 mm^2^, matrix size = 192 × 192, 160 slices, slice thickness = 1 mm, generalized autocalibrating partial parallel acquisition (GRAPPA) acceleration factor = 2, acquisition time = 4 min 8 s). MR angiography (MRA) was collected using a 3D time-of-flight (TOF) sequence (TR/TE/FA = 22 ms/3.86 ms/15°, FOV = 200 × 200 mm^2^, matrix size = 256 × 256, slice thickness = 0.62 mm, GRAPPA acceleration factor = 2, acquisition time = 3 min 32 s). MRA acquisition covered the major arteries including the circle of Willis (slab thickness = 80 mm) and a saturation band was applied over the acquisition block to limit venous contamination. MR venography (MRV) was collected using a 2D TOF sequence (TR/TE/FA = 18 ms/3.79 ms/60°, FOV = 220 × 220 mm^2^, matrix size = 256 × 256, slice thickness = 3.0 mm, GRAPPA acceleration factor = 2, acquisition time = 2 min 44 s). Echo-planar spectroscopic imaging (EPSI) with manual B_0_ shimming was acquired and used for MR thermometry as previously described^46,47^ (TR1/TR2/TE = 1551/511/17.6 ms, FA = 71°, FOV = 280 × 280 × 180 mm^3^, k-space sampling = 500 points with 1250 Hz spectral bandwidth, 50 × 50 voxels, 18 slices, nominal resolution = 5.6 × 5.6 × 10 mm^3^, interpolated resolution = 4.4 × 4.4 × 5.6 mm^3^ (64 × 64 × 32 data points), acquisition time = 15 min 17 s). The same center slice position and orientation were used for both the EPSI and T1-weighted image acquisition to facilitate image registration. A saturation band was placed over the sinuses and cavity regions to avoid contamination of neighboring voxels (Fig. S1). Lipid inversion nulling (TI = 198 ms) was performed, and interleaved non-water suppressed and water-suppressed (using the chemical shift selective suppression sequence (suppression bandwidth = 35 Hz)) scans were acquired. Axillary temperature was recorded at three time points during the EPSI sequence (at the start of the scan, at 8 min, at the end of the scan) using a fiber optic temperature sensor (OTG-MPK5, Opsens) placed underneath the left arm. The average axillary temperature for each subject was used to estimate the effect of using subject-specific inlet arterial temperatures in the model. Inlet arterial temperatures for each subject were approximated as the pulmonary artery temperature, calculated using the previously published relationship between axillary (ax) and pulmonary artery (PA) PA temperatures (T_PA_ = T_ax_ + 0.47 °C)^48^.

### Whole brain MR thermometry

MR-measured brain temperature maps were generated using the metabolite imaging and data analysis system (MIDAS)^47,49^. Pre-processing included eddy current correction, zero-order phasing, and correction for B_0_ field inhomogeneity, followed by Fourier transform, spectral denoising using principal component analysis, and spectral fitting using FITT 2.1 in MIDAS^50^. Temperature maps were calculated using the chemical shift difference between water and *N*-acetylaspartate (NAA), correcting for frequency differences between gray matter (GM) and white matter (WM)^46,51^. Four criteria for spectral quality control were used and voxels meeting all four were included in the final analysis: (1) water linewidth < 18 Hz, (2) metabolite linewidth < 8 Hz, (3) Cramer–Rao lower bounds for the creatine peak < 15%, and (4) passing a spectral outlier test^49,51^. Temperature maps generated with MIDAS (4.4 × 4.4 × 5.6 mm^3^) were resampled using a 4th degree B-spline interpolation^52–54^ and co-registered using a voxel-to-voxel affine transformation (with normalized mutual information) to T1-weighted image space (1.3 × 1.3 × 1.0 mm^3^)^55,56^ in SPM 12.

### Biophysical modeling of brain temperature

**Figure 1:**
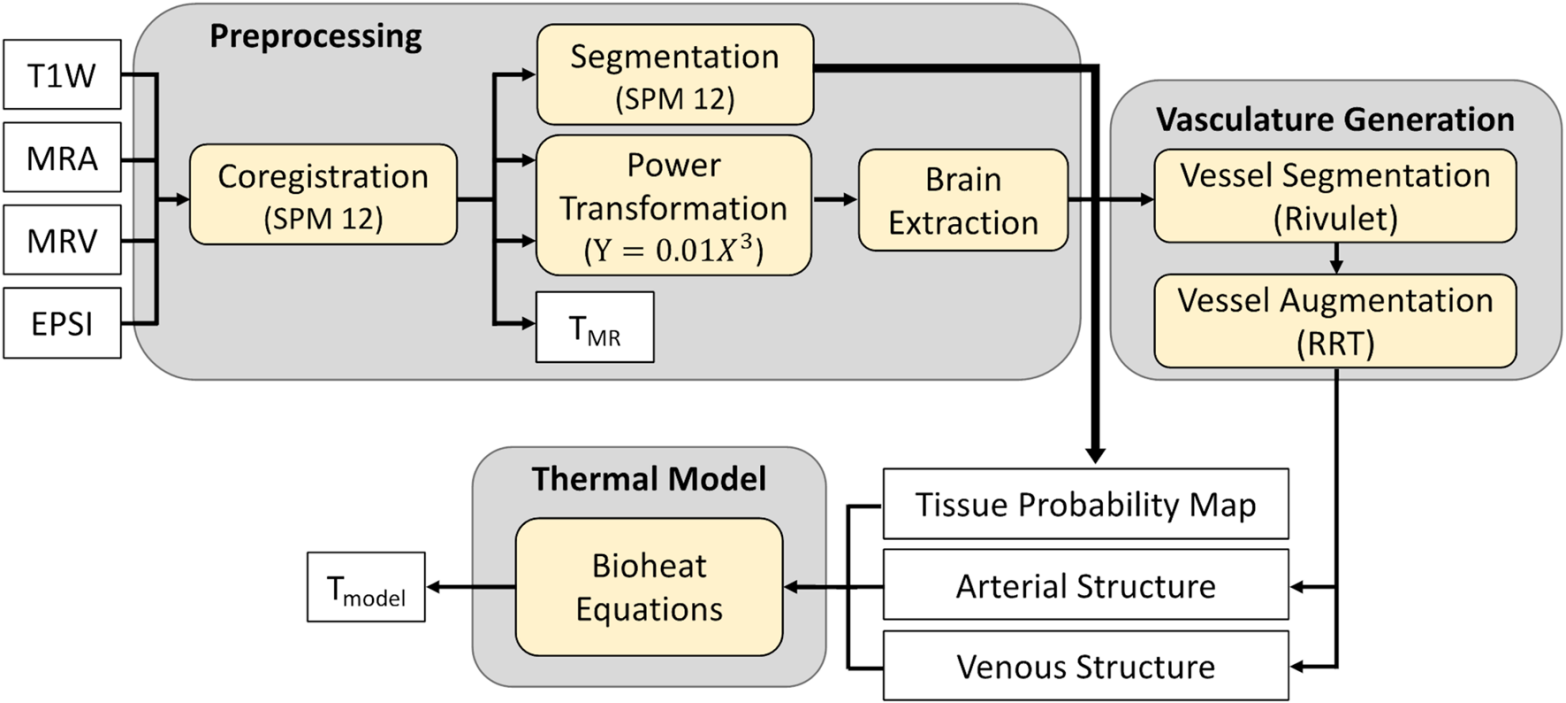
Overview of our subject-specific biophysical model used to generate personalized whole brain temperature maps. Input MR data (T1W, MRA, MRV) is acquired from each subject and used in the model. Model-predicted temperatures are compared to MR-measured temperatures acquired independently with MR thermometry using EPSI. *T1W* T1-weighted MR images, *MRA* MR angiography, *MRV* MR venography, *EPSI* echo planar spectroscopic imaging, *RRT* rapidly exploring random tree, *T*_*model*_ Model-predicted brain temperature, *T*_*MR*_ MR-measured brain temperature.

Whole brain simulated temperature maps were generated using our biophysical model with MR input data for each individual subject as previously described in detail^46^. Briefly, intra-domain (within blood vessels and within tissue voxels) and inter-domain (between vessels and voxels) cerebral blood flow (CBF) rates are calculated using bioheat transfer equations. The values for heat generation (e.g., metabolic rates) and thermophysical properties (e.g., tissue density, thermal conductivity, specific heat of tissue and blood) were the same as previously reported^45^. These properties were aggregated from early bioheat transfer models^57–59^ and derived from in vivo or ex vivo experiments. Voxel-wise physical properties were then calculated by applying these a priori data to MR-derived tissue probability maps (TPMs) generated from segmented T1-weighted images^45,46^. Brain temperature is calculated by solving the discretized form of the governing equations as described in our previous pilot study^46^. To combine tissue and vascular information in the same model, MRA and MRV data were also transformed to T1-weighted image space in SPM 12 (see “Whole brain MR thermometry” section)^55,60^, followed by a power-law transformation to enhance image contrast. Automatic vessel segmentation was facilitated with Rivulet^61,62^. Due to the limited spatial resolution of MRA and MRV, additional fine blood vessel segments were augmented to the originally segmented vessel tree using a rapidly exploring random tree (RRT) algorithm as previously reported^46,63^. It was assumed that more dense vasculature will exist where higher CBF is observed, and branched nodes were generated based on the CBF map estimated from TPMs and a priori CBF values in gray and white matter. Input parameters were selected as follows: inlet arterial temperature was fixed to 36.8 °C (the median between carotid artery temperature [36.6 °C]^64^ and brain tissue temperature [37.0 °C]^65^ in humans) or approximated for each subject using pulmonary artery temperature (see “Study participants and MR acquisition” section); and terminal capillary diameter was set to 7 μm based on previous studies^66,67^. A schematic of the input data generation and modeling procedure is shown in Fig. 1. All input data (T1-weighted images, MRA, and MRV) were used to determine model-predicted temperature independently of the MRS (EPSI) data used for MR-measured temperature calculations.

### Regional analysis

Regional analysis was conducted using both subject-specific and group-averaged temperature maps. For regional comparisons at the subject level (in resampled T1-weighted image space, 1 mm isotropic voxels), cortical and subcortical regions were parcellated in FreeSurfer 6.0 (http://surfer.nmr.mgh.harvard.edu/) using the Desikan–Killiany atlas^68^ and Gaussian classifier atlas^69^. Ten cortical regions (left and right frontal lobes, temporal lobes, parietal lobes, occipital lobes, and insula) and twelve subcortical regions (left and right cerebral WM, cerebellar WM, cerebellar cortex, thalamus, putamen, and pallidum) were used for analysis. Atlas regions with any dimension smaller than the original EPSI voxel size (4.4 × 4.4 × 5.6 mm^3^) were excluded (e.g., cingulate, hippocampus, etc.) to reduce partial volume effects.

To facilitate group-averaged comparisons, MR-measured temperature maps for all subjects were registered to Montreal Neurological Institute (MNI)152 space (2 mm isotropic voxels) in MIDAS^70,71^. Model-predicted temperature maps generated in T1-weighted image space were transformed to MNI152 space in SPM 12^55,60^. MNI-registered temperature maps (both MR-measured and model-predicted) were smoothed using a 3D Gaussian filter (kernel size = 5 × 5 × 5 voxels, sigma = 1) in the spatial domain, averaged for all subjects, and segmented into 8 lobar regions (right and left frontal, parietal, temporal, and occipital lobes (Fig. S2)) using the built-in segmentation tools in MIDAS^72,73^.

### Comparison of model-predicted and MR-measured brain temperatures

As in our previous work^46^, we used a threshold of 0.8 °C based on the uncertainty of absolute temperature measurements in a phantom study performed using the same scanner^28^. For voxels satisfying all four criteria for spectral quality control (see “Whole brain MR thermometry” section), the percentage of within-threshold voxels was calculated for each subject. As the range of temperatures in model predictions was narrower than that of MR-measured temperatures, Z-scores were calculated as Z_*i*,*j*_ = [T_*i*,*j*_ − mean(T_*i*,*j*_)]/SD(T_*i*,*j*_)), with T_*i*,*j*_ being one of 660 data points in region *i* of subject *j* for either MR-measured or model-predicted temperatures, and used for comparison. Bland–Altman plots were constructed for all cortical and subcortical regions and used to visually compare model-predicted and MR-measured temperatures. Values are reported throughout as the mean ± SD unless otherwise noted.

### IRB statement

This study was approved by the Emory Institutional Review Board, and all subjects provided written informed consent prior to participation.

## Results

### Voxel-wise agreement between model-predicted and MR-measured temperatures

Of voxels meeting all criteria for spectral quality (~ 50% of all EPSI voxels for all subjects), 97.9 ± 1.6% were within the threshold for agreement (0.8 °C) between model-predicted and MR-measured temperatures (range 94.4 to > 99.9% across all subjects). Spectral quality maps and comparison of model-predicted and MR-measured temperatures are shown in Fig. S3. Most voxels that did not meet criteria for spectral quality were those suppressed during acquisition with saturation bands (e.g., near the sinuses and cerebellum) to avoid contaminating neighboring voxels. Inter-subject variability of whole brain temperature from model-predictions was smaller (SD = 0.01 °C, confidence interval = [37.02 °C, 37.06 °C]) than MR measurements (SD = 0.09 °C, confidence interval = [36.86 °C, 37.21 °C]).

### Regional agreement between model-predicted and MR-measured temperatures

**Figure 2:**
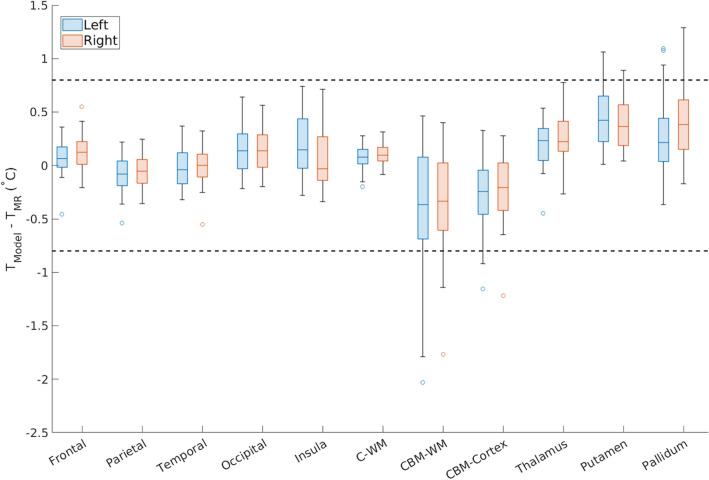
Boxplots of temperature differences between model-predicted and MR-measured temperatures for all subjects. Horizontal dashed lines indicate the agreement threshold of 0.8 °C, and mean differences for all regions were within the threshold. The boxes represent the interquartile range (IQR), the center line represents the median, and whiskers are within 1.5 times the IQR. Outliers are shown as open circles. *T*_*Model*_ model-predicted brain temperature, *T*_*MR*_ MR-measured brain temperature, *Frontal* frontal lobe, *Parietal* parietal lobe, *Temporal* temporal lobe, *Occipital* occipital lobe, *C-WM* cerebral white matter, *CBM-WM* cerebellar white matter, *CBM-Cortex* cerebellar cortex. Raw data is shown in Table S1.

**Figure 3:**
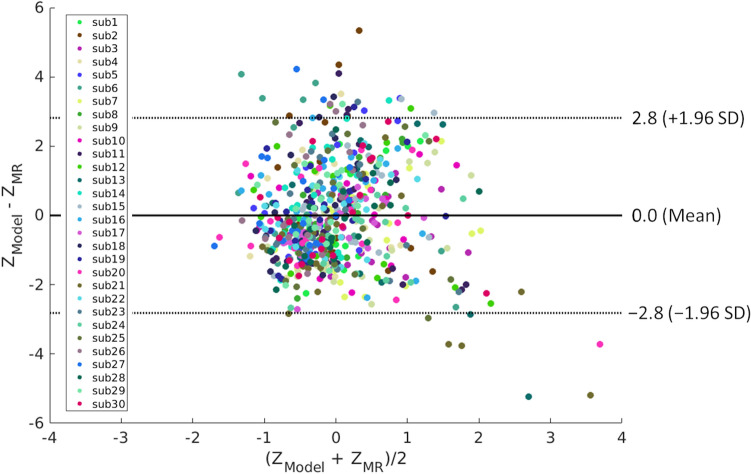
Bland–Altman plot demonstrating agreement between model-predicted and MR-measured temperatures for all subjects and 22 cortical and subcortical regions. Data points are color-coded by subject. *Z*_*model*_ Z-scores of model-predicted brain temperatures, *Z*_*MR*_ Z-scores of MR-measured brain temperatures, *SD* standard deviation, *sub* subject.

As voxel-wise agreement was high, regional analysis was performed to facilitate further comparison of thermal gradients. For all regions, mean absolute differences were within the agreement threshold of 0.8 °C (Fig. 2, Table S1). Mean differences in model-predicted and MR-measured temperatures among cortical regions were all within the agreement threshold and ranged from 0.13 °C in the left frontal lobe to 0.27 °C in the left insula. In subcortical regions, differences ranged from 0.11 °C in left cerebral WM and 0.54 °C in left cerebellar WM. Unlike cortical regions, several subcortical regions (left/right cerebellar WM, cerebellar cortex, putamen, and pallidum) had maximum absolute temperature differences exceeding the agreement threshold of 0.8 °C. Left hemispheric temperatures were 0.03 °C and < 0.01 °C higher than the right hemisphere from model-predictions and MR measurements, respectively. Subcortical regions were 0.09 °C and 0.07 °C higher than cortical regions for model-predicted and MR-measured temperatures, respectively. Regional averages of MR-measured temperatures spanned a larger range (35.85–39.18 °C) compared to model-predictions (36.95–37.28 °C) for all subjects. Across all regions and subjects, the majority (94.4%) of regional temperature-derived Z-scores between model-predictions and MR measurements were within the limits of agreement in Bland–Altman analysis (Fig. 3). While minimal bias was observed across subjects (Fig. 3), bias was observed in some brain regions (Fig. S4). Model-predicted and MR-measured temperatures were largely similar in cerebral WM. MR-measured temperatures were higher than model-predictions in cerebellar regions, and the opposite trend was observed in putamen and pallidum regions.

### Global brain temperature patterns

**Figure 4:**
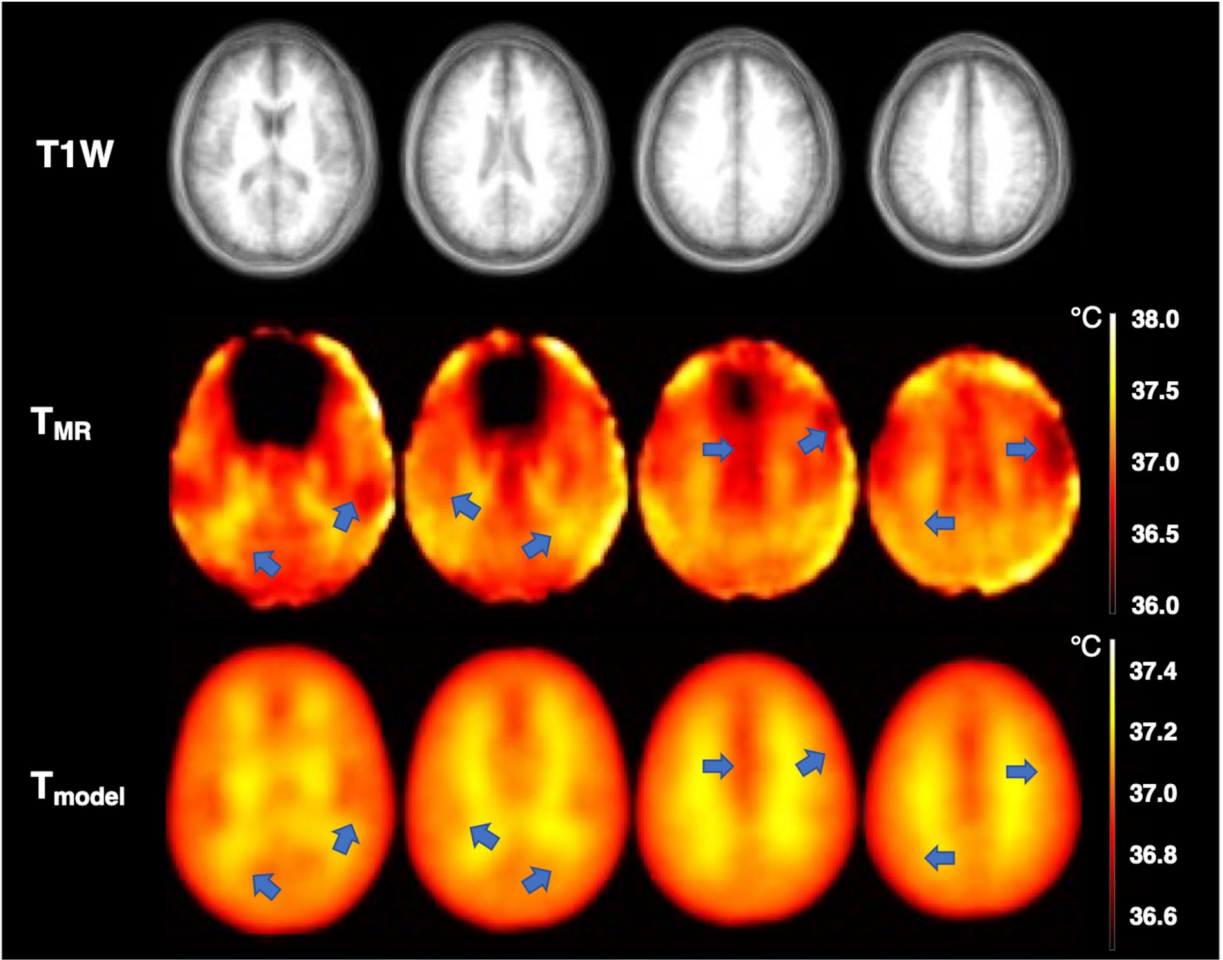
Comparison of group-averaged brain temperature maps from MR measurements and model-predictions for multiple axial slices. The first row shows the anatomical images corresponding to the same axial slice as the temperature maps. While spatial variation exists, the deep brain and subcortical white matter is higher in both model and measurement (highlighted in blue arrows). MR-measured temperatures range from [36.0 °C, 38.0 °C], while model-predicted temperatures range from [36.5 °C, 37.5 °C]. *T1W* T1-weighted image, *T*_*MR*_ MR-measured brain temperatures, *T*_*model*_ model-predicted brain temperatures.

**Figure 5:**
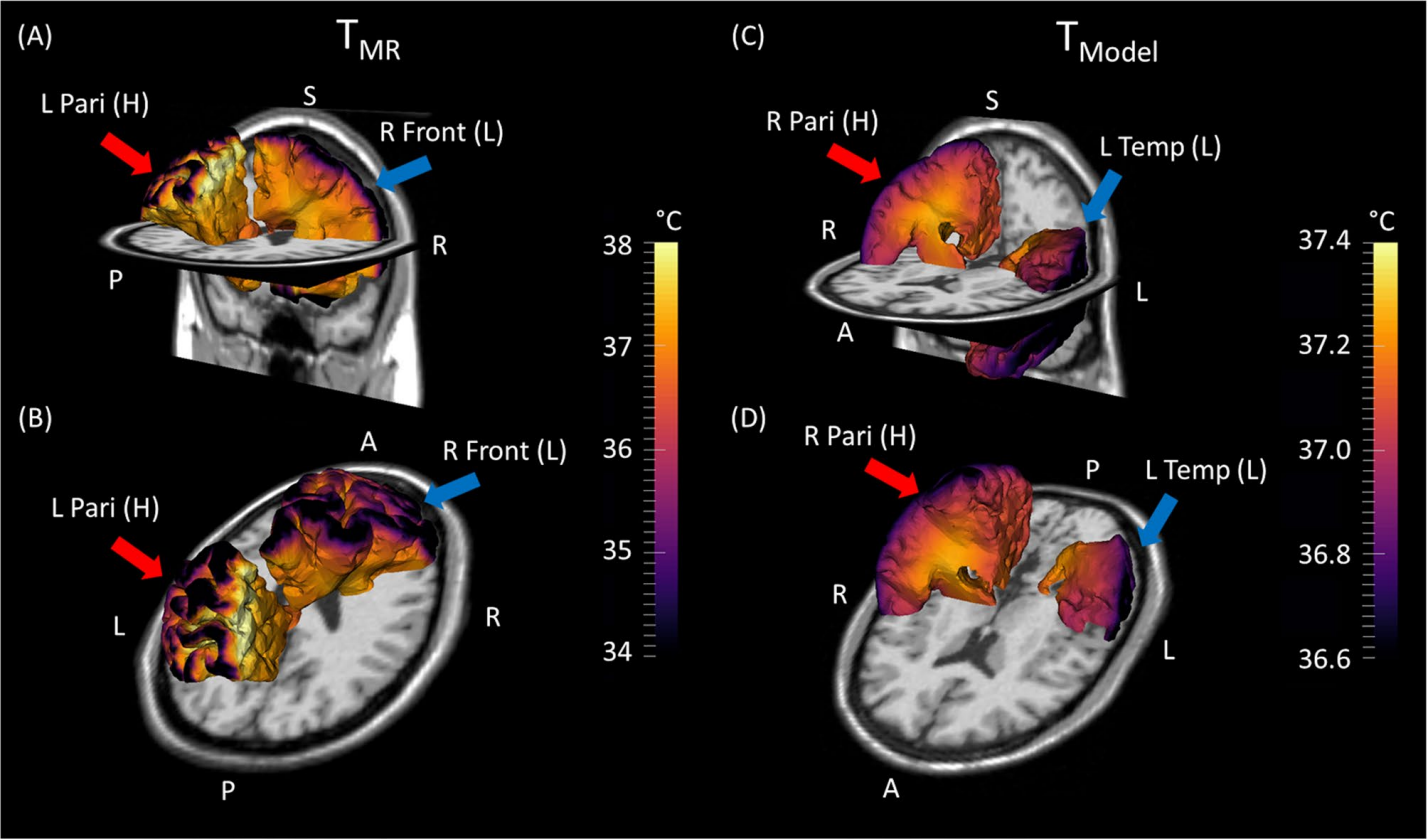
Lobar-scale 3D images overlaid with (**A,B**) MR-measured and (**C,D**) model-predicted brain temperature maps. Lobes with the highest average temperature (i.e., left parietal lobe [T_MR_] and right parietal lobe [T_Model_]) are highlighted with red arrows. Lobes with the lowest average temperature (i.e., right frontal lobe [T_MR_] and left temporal lobe [T_Model_]) are highlighted with blue arrows. MR-measured temperatures range from [34.0 °C, 38.0 °C], while model-predicted temperatures range from [36.6 °C, 37.4 °C]. *T*_*MR*_ MR-measured temperature, *T*_*Model*_ model-predicted temperature, *L* left, *R* right, *Pari* parietal lobe, *Front* frontal lobe, *Temp* temporal lobe, *(H)* highest temperature lobe, *(L)* lowest temperature lobe.

To characterize global patterns in healthy brain temperature, whole brain model-predicted and MR-measured temperature maps in standardized MNI-space were investigated. While voxel-wise and regional comparisons showed good agreement at the subject level, global analysis facilitates characterization of expected biophysical trends, e.g., as a function of tissue type and brain region. Qualitatively, subcortical WM regions tend to have relatively higher temperatures compared to other regions in both MR-measured and model-predicted temperature maps (Fig. 4). Quantitatively, the lowest and the highest MR-measured temperatures were observed in the right frontal lobe (36.87 °C) and left parietal lobe (37.12 °C), respectively (Fig. 5A,B). From model-predictions, the lowest and the highest temperatures were observed in left temporal lobe (37.06 °C) and right parietal lobe (37.10 °C), respectively (Fig. 5C,D). The maximum absolute difference between MR-measured and model-predicted temperatures was observed in the right frontal lobe with a value of 0.22 °C, likely due to suppression of signal in portions of the frontal lobe (see “Methods” section). The minimum absolute difference was observed in the left temporal lobe (0.01 °C), indicating a region with high consistency between model-predictions and MR measurements. Average venous temperatures in the internal jugular vein from model-predictions was 37.0 °C across all subjects, 0.2 °C higher than the input arterial temperature (36.8 °C) used in the model. Mean arterial temperatures calculated from experimentally-measured axillary temperatures for all subjects were 36.80 ± 0.55 °C, similar to the fixed input arterial temperature (36.8 °C) used in the model. The use of a subject-specific inlet arterial temperature (estimated using pulmonary arterial temperature; see “Study participants and MR acquisition” section) in the model resulted in a maximum bias for whole brain temperature of ~ 0.5 °C and did not alter spatial thermal gradients.

## Discussion

High overall agreement was observed between model-predictions and MR measurements, however, variations in temperature ranges and spatial temperature patterns were observed. Regional analysis revealed similar model-predicted and MR-measured temperatures across most brain regions, with the lowest difference (0.11 ± 0.07 °C) observed in left cerebral white matter. While mean differences between model-predictions and MR measurements were within the agreement threshold (0.8 °C) for all regions, the largest differences in subject-specific regional analyses were observed in left and right cerebellar WM (Table S1) likely due to portions of the suppression band covering much of the cerebellum.

Maudsley et al. previously reported reproducibility errors in regional (lobar-scale) MR-measured temperatures of 0.2 °C^51^. From our group-averaged, lobar-scale comparisons, the maximum absolute difference between model-predictions and MR measurements was 0.22 °C (right frontal lobe). This suggests at the lobar-scale, agreement between MR measurements and model-predictions is on the same scale as reproducibility errors in whole brain MR thermometry. Within each method (i.e., MR measurements and model-predictions), regional differences were observed. The highest temperatures were observed in parietal lobes for both MR measurements and model-predictions. For MR measurements, higher temperatures were observed in the left parietal lobes (Fig. 5A,B); further exploration of regional differences is warranted. The lowest measured temperatures in the right frontal lobe (Fig. 5A,B) are attributed, at least in part, to the limited number of voxels in the frontal lobe as many were excluded due to the saturation band. For model-predictions, highest temperatures were observed in the right parietal lobe (Fig. 5C,D) as group-averaged GM/WM ratios were lowest in parietal lobes, resulting in the lowest CBF^74,75^ and less heat removal by blood circulation. Similarly, the lowest model-predicted temperatures observed in the left temporal lobe (Fig. 5C,D) can also be explained by the highest GM/WM ratio in this region.

Higher temperatures in subcortical regions were observed compared to cortical regions for both model-predictions and MR measurements, consistent with prior reports^13,46,51,76^. Cortical regions are related to high-level function such as decision-making or sensing the surrounding environment and have more dense arterial and venous structure, resulting in higher CBF with more cooling. Increased conduction near the skull due to lower ambient temperature outside the head also results in lower temperatures in superficial cerebral sites and relatively higher deep brain temperatures^77^. MR-measured temperatures were also higher in the left hemisphere compared to the right hemisphere by 0.03 °C, consistent with prior reports observing 0.03 °C higher temperatures in the left hemisphere^51^. For model-predictions, average internal jugular vein temperature was 37.0 °C across all subjects, 0.2 °C higher than the input arterial temperature (36.8 °C) in our model. Previous literature reported a ~ 0.3 °C temperature difference between arterial and venous temperatures, similar to our findings^5,78^. As heat dissipation in the brain is largely due to conduction and heat transfer from tissue to cooler incoming blood, this gradient in vessel temperatures is expected particularly in the healthy brain.

Temperature differences between model-predictions and MR measurements were primarily attributed to challenges in MR thermometry acquisition in some regions (e.g., susceptibility near the sinus cavity, suppression band placement, etc.) and the narrow range of model-predicted temperatures compared to MR measurements. Susceptibility artifacts can quickly deteriorate spectral quality and reduce the accuracy of chemical shift MR thermometry^51,79,80^. MR-measured temperatures at the brain periphery in some regions are relatively higher than the corresponding model-predicted temperatures, attributed in part to susceptibility artifacts in experimental thermometry at the tissue boundaries. While manual shimming and adjustment of suppression bands were both applied in this study to minimize susceptibility, improvements in MR spectroscopy acquisition may alleviate some of these challenges. The suppression band resulted in partial signal loss in the frontal lobe as described in previous work^47,51^, but reduces further spectral contamination from susceptibility artifacts or field inhomogeneity (particularly for echo planar acquisition) from the sinuses or other cavities. We acknowledge the voxel size used in EPSI acquisition may lead to some partial volume effects in smaller regions; however, atlas-defined regions with dimensions smaller than the voxel size were excluded to minimize this as much as possible. While reliability of the 3D EPSI sequence used for whole brain MR thermometry was not specifically investigated in the current work, many prior studies have evaluated its reproducibility both for metabolite quantification^81,82^ and chemical shift thermometry^51,83,84^. Maudsley et al. used Monte-Carlo simulations to determine SD of the frequency measurement with EPSI is 0.0018 ppm (corresponding to ~ 0.2 °C) with a maximum error of 0.006 ppm (corresponding to ~ 0.6 °C)^51^. Zhang et al. assessed the SD of regional temperature in healthy volunteers as a metric of repeated measurement accuracy, reporting a mean (range) of 0.4 (0.3–0.8) °C across the entire brain. Intrasubject coefficient of variation (CV) was 0.9% for brain temperature measurement using creatine as a temperature reference using the 3D EPSI sequence^83^. Similarly, Sharma et al. evaluated reproducibility of EPSI and observed stable temperature variations with mean CV across repeated measures of 1.9%^84^. Despite the limitations, these data support the use of EPSI as an experimental thermometry method suitable for comparison with biophysical model predictions. Finally, subject-specific brain tissue and vessel structure was used in our model; however, the same inlet arterial temperature was used for all subjects which may be partly responsible for the relatively small variation (SD = 0.01 °C) across subjects compared to MR measurements (SD = 0.09 °C).

Future work will address several limitations. While body temperature (e.g., axillary) is not an ideal surrogate for arterial temperature, the use of body temperature for each subject in our model as a boundary condition may be necessary to account for individual variations, particularly as brain and body temperature are correlated in healthy mammals^13–16^. MRA and MRV, while relatively fast, are not optimal methods for constructing vessel structure and calculating CBF. Refinement of vessel distribution with more accurate measurements such as computed tomography angiography or acquisition at higher field strengths (e.g., 7 T) may be necessary. Similarly, direct measurements of blood flow and perfusion using, e.g., arterial spin labeling, may further improve our model. The biophysical model could also be further improved by incorporation of momentum conservation for blood flow in all simulation domains, as well as through better assessment of the input parameters and boundary conditions that are used for predicting the brain thermal behavior of a given individual. In the case of rigorous momentum conservation in the model, some thermophysical parameters which determine the thermal resistance of blood vessels and tissue may impact thermal distribution. Robust sensitivity analysis to input parameters is an immediate next step. While experimental thermometry acquired with the whole brain EPSI sequence were consistent with previous reports^51^, artifacts near the sinuses or other cavities, field inhomogeneity from the echo planar acquisition, and long scan times are limitations of this method. The experimental temperature maps were resampled and transformed to T1-weighted image space to facilitate comparisons between MR measurements and model-predictions, and some uncertainty may be introduced at the voxel-level. Finally, as the model was evaluated with data from healthy volunteers, future studies will investigate the agreement between model-predictions and MR measurements using patient data (e.g., chronic cerebrovascular disease, cardiac arrest, severe traumatic brain injury, etc.) and determine the required accuracy and resolution for the use of brain temperature as a non-invasive marker for diagnosis or treatment planning.

## Conclusions

Personalized model-predicted brain temperature maps for 30 healthy subjects were compared with whole brain MR thermometry, and agreement in both voxel-wise and regional temperature distributions were observed. While differences exist, we expect the combined and complementary use of model-predictions and MR measurements is an emerging paradigm for further development of brain temperature as a promising biomarker for prognostication and treatment monitoring.

## Supplementary Information

Supplementary Information.

## Data Availability

Raw data will be made available upon request to the corresponding author and after a resource sharing agreement is in place, as required by the authors’ institutions.
