# Supplementary material for: Comparisons of healthy human brain temperature predicted from biophysical modeling and measured with whole brain MR thermometry
Source: Sci Rep. 2022 Nov 11;12:19285. doi: 10.1038/s41598-022-22599-x (PMC9652378; doi:10.1038/s41598-022-22599-x)
Supplement: Supplementary file 1 — Supplementary Information. [file 41598_2022_22599_MOESM1_ESM.docx]

**Supplementary Materials for**

**Comparisons of healthy human brain temperature predicted from biophysical modeling and measured with whole brain MR thermometry**

Dongsuk Sung, Benjamin B. Risk, Peter A. Kottke, Jason W. Allen, Fadi Nahab,

Andrei G. Fedorov, and Candace C. Fleischer*

*Corresponding author email: [candace.fleischer@emory.edu](mailto:candace.fleischer@emory.edu)

**This file includes:**

**Figure S1.** Screenshot of the echo planar spectroscopic imaging (EPSI) acquisition volume overlaid onto the sagittal 3D localizer image for one subject.

**Figure S2.** Lobar-scale regions used in analysis in the (A) axial, (B) sagittal, and (C) coronal views.

**Figure S3.** (A) Spectral quality maps for all subjects. (B) Voxel-wise comparison of model-predicted and MR-measured temperatures for all subjects in the same axial slice shown in (A).

**Figure S4.** Bland-Altman plot of all 22 regions across all 30 subjects color-coded by regions.

**Table S1**. MR-measured and model-predicted temperatures for 22 regions (10 cortical and 12 subcortical).


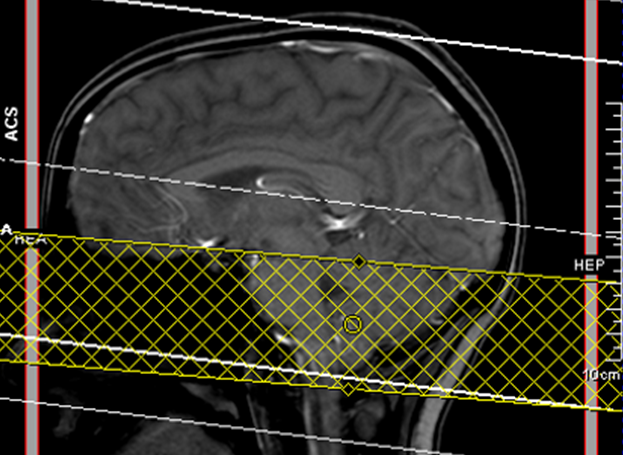


**Figure S1.** Screenshot of the echo planar spectroscopic imaging (EPSI) acquisition volume overlaid onto the sagittal 3D localizer image for one subject. The field of view is indicated by white horizontal solid lines and saturation bands to suppress the sinuses and other cavities are shown as yellow cross-hatched grids. Portions of the frontal lobe were also suppressed to avoid contamination of neighboring voxels.

**
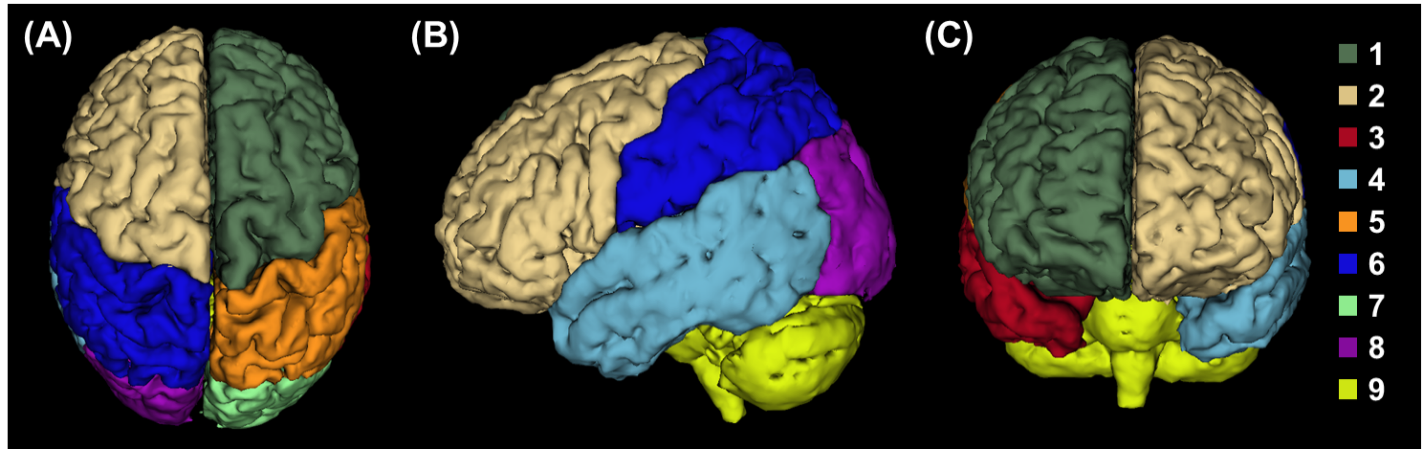
**

**Figure S2.** Lobar-scale regions used in analysis; (A) axial, (B) sagittal, and (C) coronal views. 1: right frontal lobe; 2: left frontal lobe; 3: right temporal lobe; 4: left temporal lobe; 5: right parietal lobe; 6: left parietal lobe; 7: right occipital lobe; 8: left occipital lobe; 9: cerebellum.


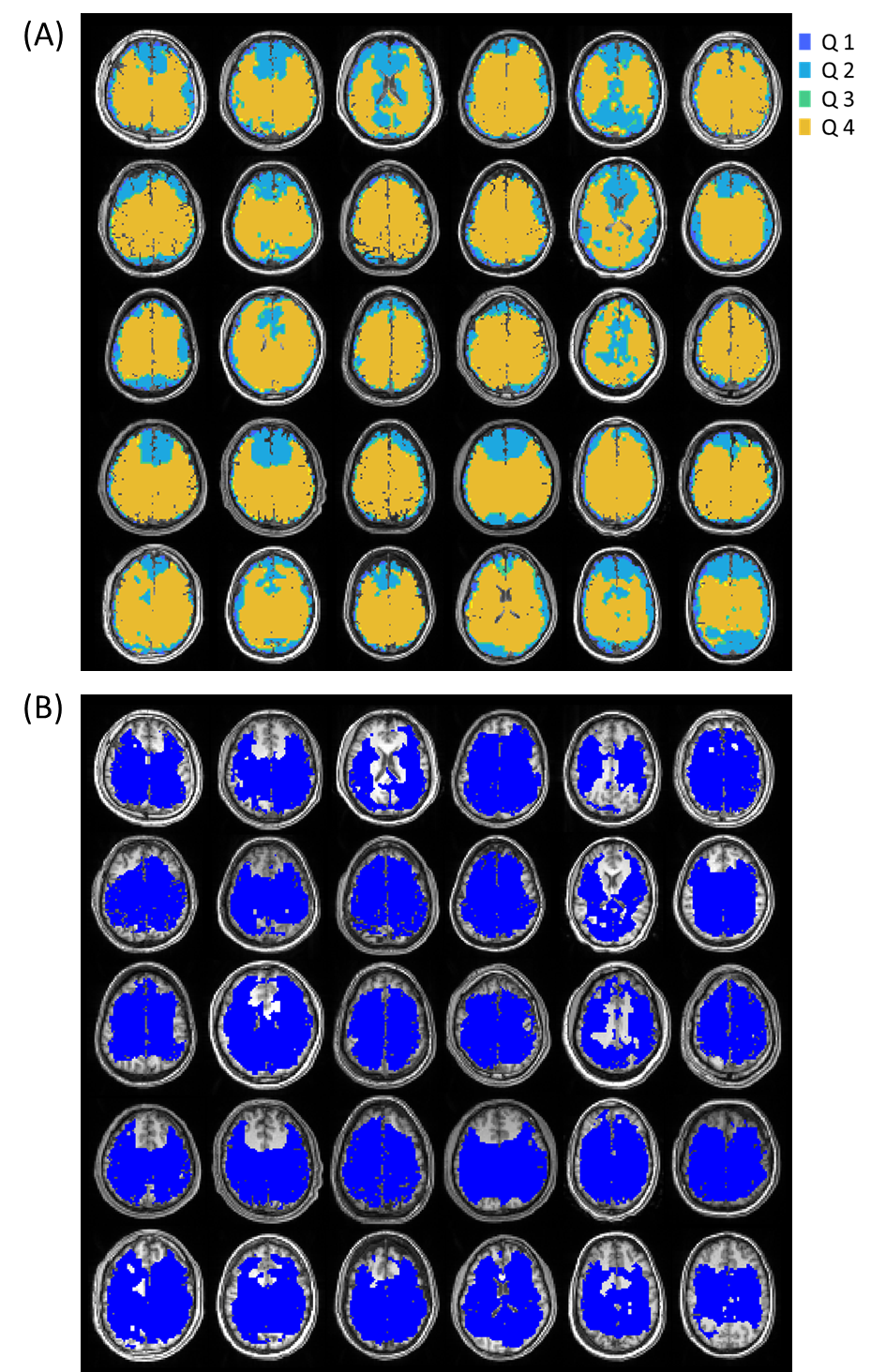


**Figure S3.** (A) Spectral quality maps for all subjects. Voxels meeting quality control criteria (Q) as described in the Methods are shown, i.e., voxels meeting all four criteria are color-coded in yellow (Q4), voxels meeting the first three criteria are color-coded in teal (Q3), etc. (B) Voxel-wise comparison of model-predicted and MR-measured temperatures for all subjects in the same axial slice shown in (A). Voxels meeting all four quality control criteria and with temperature differences within the agreement threshold (0.8 °C) are shown in blue (97.9±1.6% for all subjects).


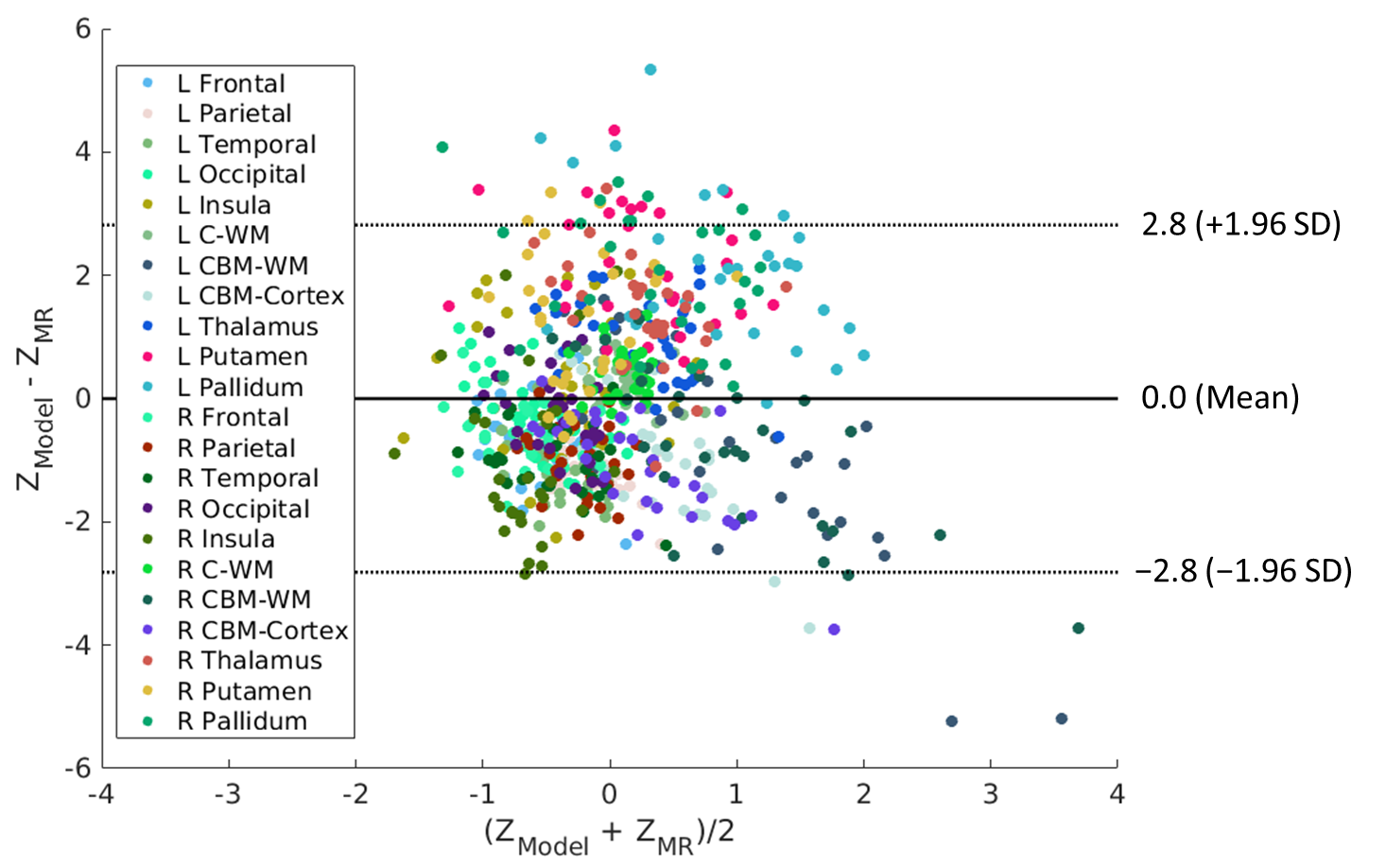


**Figure S4.** Bland-Altman plot demonstrating agreement between model-predicted and MR-measured temperatures for all subjects and 22 cortical and subcortical regions. Data points are color-coded by region. Z_Model_: Z-scores of model-predicted brain temperature; Z_MR_: Z-scores of MR-measured brain temperature; L: left; R: right; Frontal: frontal lobe; Parietal: parietal lobe; Temporal: temporal lobe; Occipital: occipital lobe; C-WM: cerebral white matter; CBM-WM: cerebellum white matter; CBM-Cortex: cerebellum cortex.

**Table S1**. MR-measured and model-predicted temperatures for 22 regions (10 cortical and 12 subcortical). Values are reported as the mean ± standard deviation.

| **Region Name** | **T_Model_** | **T_MR_** | **Diff** | **\|Diff\|** | **Max \|Diff\|** |
| --- | --- | --- | --- | --- | --- |
| L Frontal | 37.03±0.02 | 36.96±0.16 | 0.07±0.16 | 0.13±0.12 | 0.46 |
| L Parietal | 37.05±0.02 | 37.13±0.16 | -0.08±0.16 | 0.13±0.12 | 0.54 |
| L Temporal | 37.03±0.02 | 37.05±0.18 | -0.02±0.18 | 0.15±0.10 | 0.37 |
| L Occipital | 37.04±0.02 | 36.89±0.23 | 0.15±0.23 | 0.21±0.18 | 0.64 |
| L Insula | 37.07±0.05 | 36.88±0.28 | 0.19±0.29 | 0.27±0.22 | 0.74 |
| L cerebral WM | 37.11±0.01 | 37.03±0.11 | 0.08±0.11 | 0.11±0.07 | 0.28 |
| L Cerebellar WM | 37.13±0.03 | 37.52±0.61 | -0.39±0.61 | 0.54±0.47 | 2.03 |
| L Cerebellar Cortex | 37.08±0.02 | 37.33±0.33 | -0.25±0.33 | 0.32±0.26 | 1.16 |
| L Thalamus | 37.13±0.03 | 36.93±0.24 | 0.20±0.23 | 0.25±0.17 | 0.54 |
| L Putamen | 37.17±0.04 | 36.74±0.29 | 0.43±0.29 | 0.43±0.29 | 1.06 |
| L Pallidum | 37.21±0.04 | 36.96±0.39 | 0.25±0.39 | 0.35±0.29 | 1.09 |
| R Frontal | 37.02±0.02 | 36.90±0.18 | 0.12±0.18 | 0.18±0.13 | 0.55 |
| R Parietal | 37.04±0.02 | 37.11±0.14 | -0.07±0.14 | 0.13±0.10 | 0.36 |
| R Temporal | 37.03±0.03 | 37.04±0.19 | -0.01±0.18 | 0.14±0.12 | 0.55 |
| R Occipital | 37.05±0.02 | 36.92±0.18 | 0.13±0.19 | 0.19±0.14 | 0.56 |
| R Insula | 37.02±0.05 | 36.97±0.25 | 0.05±0.28 | 0.23±0.17 | 0.72 |
| R cerebral WM | 37.11±0.02 | 37.00±0.09 | 0.11±0.10 | 0.12±0.09 | 0.31 |
| R Cerebellar WM | 37.12±0.04 | 37.47±0.51 | -0.35±0.50 | 0.46±0.39 | 1.77 |
| R Cerebellar Cortex | 37.07±0.02 | 37.30±0.32 | -0.23±0.32 | 0.29±0.26 | 1.22 |
| R Thalamus | 37.15±0.03 | 36.87±0.25 | 0.28±0.25 | 0.31±0.21 | 0.78 |
| R Putamen | 37.11±0.04 | 36.72±0.23 | 0.39±0.25 | 0.39±0.25 | 0.89 |
| R Pallidum | 37.17±0.05 | 36.79±0.33 | 0.38±0.32 | 0.40±0.30 | 1.29 |
| L: left; R: right; WM: white matter; T_Model_: model-predicted temperature; T_MR_: MR-measured temperature; Diff: mean T_Model_ −T_MR_ for all subjects; \|Diff\|: absolute difference between T_Model_ and T_MR_ for all subjects. | | | | | |
